# Genetic and phenotypic insights into *Cyberlindnera jadinii* as a promising yeast for industrial biotechnology

**DOI:** 10.1093/g3journal/jkaf145

**Published:** 2025-06-30

**Authors:** Junyuan Wu, Shinsuke Ohnuki, Élie Teyssonnière, Taishi Yasukawa, Naohisa Masuo, Joseph Schacherer, Yoshikazu Ohya

**Affiliations:** Mitsubishi Corporation Life Sciences Limited, Tokyo Takarazuka Building 14F, 1-1-3 Yurakucho, Chiyoda-ku, Tokyo 100-0006, Japan; Department of Integrated Biosciences, Graduate School of Frontier Sciences, University of Tokyo, Bldg. FSB-101, 5-1-5 Kashiwanoha, Kashiwa, Chiba Prefecture 277-8562, Japan; Unité Mixte de Recherche 7156, Department of Genetics, Genomics and Microbiology, University of Strasbourg - Centre National de la Recherche Scientifique, 28 rue Goethe, 67083 Strasbourg, France; Mitsubishi Corporation Life Sciences Limited, Tokyo Takarazuka Building 14F, 1-1-3 Yurakucho, Chiyoda-ku, Tokyo 100-0006, Japan; Mitsubishi Corporation Life Sciences Limited, Tokyo Takarazuka Building 14F, 1-1-3 Yurakucho, Chiyoda-ku, Tokyo 100-0006, Japan; Unité Mixte de Recherche 7156, Department of Genetics, Genomics and Microbiology, University of Strasbourg - Centre National de la Recherche Scientifique, 28 rue Goethe, 67083 Strasbourg, France; Institut Universitaire de France (IUF), 103 boulevard Saint-Michel, Paris 75005, France; Department of Integrated Biosciences, Graduate School of Frontier Sciences, University of Tokyo, Bldg. FSB-101, 5-1-5 Kashiwanoha, Kashiwa, Chiba Prefecture 277-8562, Japan; Department of Science and Technology Innovation, Nagaoka University of Technology, 1603-1 Kamitomioka, Nagaoka, Niigata 940-2188, Japan

**Keywords:** *Cyberlindnera jadinii*, WGS, morphology, phenotypic diversity, fitness, industrial yeast

## Abstract

*Cyberlindnera jadinii* is a nonmodel yeast species recognized for its robust growth under diverse stress conditions, making it a promising candidate for various industrial applications. Here, we performed genome sequencing for genetic analysis and high-throughput phenotypic assays to examine 20 wild strains, focusing on the diversity of their genome, morphology, and fitness traits. We found that *C. jadinii* comprises a mixed population of diploid and triploid cells, with the latter exhibiting significantly larger cell sizes than the former, suggesting that ploidy plays a crucial role in determining cell morphology. In contrast, fitness traits, including growth under 24 different conditions, were more closely associated with SNP-defined lineages than with ploidy. *Cyberlindnera jadinii* exhibited a genetic diversity (*π* = 18 × 10^−3^) four times higher than that of *Saccharomyces cerevisiae*, possibly reflecting genetic diversity shaped by history and environment. The heterologous expression of the *CjFPS1* homolog in *S. cerevisiae* enhanced its growth on glycerol and acetate, underscoring its conserved metabolic function in carbon utilization. Overall, our findings not only reveal the rich genotypic and phenotypic diversity of *C. jadinii* but also provide new insights into its potential applications as a robust industrial yeast for diverse biotechnological processes.

## Introduction

*Cyberlindnera jadinii* (historically referred to as Torula yeast and previously classified under names such as *Torula utilis*, *Hansenula jadinii*, *Candida utilis*, and *Pichia jadinii*) has undergone multiple taxonomic reclassifications over time (Barnett 2004; Sousa-Silva *et al*. 2021). Beyond its complex taxonomic history, *C. jadinii* has been widely utilized as a source of single-cell protein (SCP), particularly in the food industry, due to its generally recognized as safe (GRAS) status (Sousa-Silva *et al*. 2021). It has been incorporated into various food products, serving as a nutritional supplement and flavor enhancer, and is also used in cosmetics and pharmaceuticals (Boze *et al*. 1992; Miura *et al*. 1999; Buerth *et al*. 2016; Li *et al*. 2024).

In addition to its established roles in food and health-related industries, *C. jadinii* holds great potential in biotechnology and sustainable bioprocessing. Unlike traditional yeasts, it can efficiently utilize nonconventional carbon sources, such as glycerol, a major byproduct of biodiesel production, and xylose, a predominant sugar in lignocellulosic biomass. This metabolic versatility makes it a promising candidate for bio-based waste valorization and carbon recycling, contributing to circular bioeconomy initiatives (Tamakawa *et al*. 2011; Bzducha-Wróbel *et al*. 2018). Furthermore, its remarkable tolerance to extreme environmental conditions, including low pH as well as heavy metal contamination, enables its application in bioremediation and robust industrial fermentation processes, where conventional yeast strains may struggle to thrive (Wang *et al*. 2015; Li *et al*. 2021).

Despite its expected usefulness, *C. jadinii* remains relatively underexplored in key areas such as genomic and functional analysis. This contrasts with well-studied yeasts like *Saccharomyces cerevisiae* (Peter *et al*. 2018), *Schizosaccharomyces pombe* (Jeffares *et al*. 2015), and *Lachancea kluyveri* (Jung *et al*. 2016). Most existing studies on *C. jadinii* have focused on only a few strains, such as CBS1600 (NRRL Y-1542) and NBRC0988 (Sousa-Silva *et al*. 2021), leaving major gaps in our understanding of its genomic and phenotypic characteristics and their diversity.

One of the major issues that remains unsolved is ploidy variation, which plays a crucial role in determining yeast phenotypes and adaptive capacities. In other yeasts, differences in ploidy have been shown to influence growth rates, stress tolerance, and metabolic capabilities, yet no systematic studies have explored this factor in *C. jadinii* (Storchova 2014; Crandall *et al*. 2023). Furthermore, large-scale genotype–phenotype association studies, which have provided valuable insights into genetic diversity, breeding strategies, and adaptive traits in model and industrial yeasts (Jung *et al*. 2016; Ohnuki *et al*. 2017), are still lacking for *C. jadinii*. The absence of such comprehensive analyses leaves fundamental gaps in our knowledge of the genetic basis of phenotypic diversity in this species. Addressing these gaps is critical for optimizing *C. jadinii*'s industrial applications and uncovering novel traits of biotechnological significance.

To bridge these knowledge gaps, various approaches have been employed in yeast research to explore genomic and phenotypic diversity. High-throughput sequencing technologies facilitate the identification of genetic variations across strains, providing insights into strain-specific adaptations and evolutionary trajectories. Meanwhile, high-throughput phenotypic screening using microcultivation assays allows for systematic evaluation of growth dynamics under diverse environmental conditions. Furthermore, advanced single-cell imaging techniques, such as CalMorph-PC(10), enable precise quantification of morphological traits, including cell size and shape, shedding light on cellular phenotypic diversity (Itto-Nakama *et al*. 2023). Functional studies of key genes, such as the *FPS1* gene, which plays a crucial role in glycerol transport and stress response, further contribute to understanding the genetic determinants of phenotypic traits (Luyten *et al*. 1995). By integrating these approaches, a comprehensive genotype–phenotype framework can be established, advancing our knowledge of how genetic diversity shapes the adaptive potential of *C. jadinii*.

In this study, we applied an integrative approach combining genome sequence analysis with high-throughput phenotypic assays to explore the genomic and phenotypic diversity of *C. jadinii.* By systematically assessing its ploidy variation, growth patterns under diverse conditions, and morphological traits, we provide new insights into the factors shaping its diversity. These findings not only enhance our fundamental understanding of *C. jadinii* biology but also provide insights into its optimization for sustainable biomanufacturing, bioremediation, and industrial fermentation processes.

## Materials and methods

### Chemicals and reagents

All chemicals and reagents used in this study were of analytical or biological grade and were commercial sourced. Unless otherwise specified, general chemicals were purchased from FUJIFILM Wako Pure Chemical Corporation (Wako, Osaka, Japan) or Sigma-Aldrich Company Limited (Sigma, Cambridge, UK).

### Strains and media

This study used 20 strains of *C. jadinii* and 28 strains of *S. cerevisiae* (Supplementary Material, Table 1 in File 1). The media used to evaluate the fitness traits of each strain included YPD medium (10 g/L yeast extract, 20 g/L polypeptone, 20 g/L dextrose) supplemented with various compounds (Supplementary Table 3 in File 1). For experiments involving alternative carbon sources, 2% dextrose in the YPD medium was replaced by other carbon sources.

For biological analyses, yeast cells were cultured in SC medium (20 g/L glucose, 1.7 g/L YNB without (NH_4_)_2_SO_4_ and amino acids, 5 g/L (NH_4_)_2_SO_4_, 2 g/L casamino acids (Becton Dickinson and Company, BD, Maryland, USA) with 2 mg/L adenine, 2 mg/L uracil, 3 mg/L histidine, and 2 mg/L tryptophan) or SPM medium (10 g/L potassium acetate). SC medium without uracil (SC-ura) was used to culture mutants carrying the *URA3* marker plasmid.

### Construction of plasmids and yeast strain

Plasmids were constructed using standard molecular cloning techniques, incorporating restriction enzyme digestion (New England Biolabs, Ipswich, MA, USA) and the In-Fusion cloning system (TaKaRa Bio, Kusatsu, Japan). A specific plasmid, pRS426-TDH3pr-(CjFPS1)-GFP-TDH3ter plasmid was designed for this study. The pRS426 is a yeast multicopy plasmid with a *URA3* selection marker. The *TDH3* promoter (TDH3pr) and *GFP-TDH3* terminator (GFP-TDH3ter) sequences were derived from the pGFP-TDH3ter-*URA3* plasmid (Yasukawa *et al*. 2024).

The *CjFPS1* gene was amplified from the genomic DNA of *C. jadinii* strain ATCC9950 and inserted between the TDH3pr and GFP-TDH3ter regions. The constructed plasmid was verified through restriction enzyme digestion and sequencing before use in yeast transformation. Transformation into *S. cerevisiae* strain BY4741 was performed using the lithium acetate protocol (Gietz and Woods 2002; Gietz and Schiestl 2007). Transformants were selected on SC-ura and validated via colony PCR (Yasukawa *et al*. 2024). Primer details are provided in Supplementary File 1, Table 2.

### Morphological trait analysis

Yeast cell morphology was analyzed using CalMorph-PC(10) as described previously (Itto-Nakama *et al*. 2023). Briefly, cells were cultured in 20 mL of YPD medium at 30°C until reaching logarithmic phase. Then, cell fixation and image acquisition were performed. For each independent cell culture, at least 200 cells were imaged. Specimens from 28 *S. cerevisiae* strains were observed under an Axio Imager phase-contrast (PC) microscope equipped with a Plan-Apochromat 100×/1.4 oil lens (Carl Zeiss, Oberkochen, Germany) and CoolSNAP HQ cooled charge-coupled device camera (Roper Scientific Photometrics, Tucson, AZ, USA). In contrast, specimens from 20 *C. jadinii* strains were observed using an all-in-one bright-field (BF) microscope (BZ-X700, KEYENCE, Osaka, Japan) equipped with a Plan-Apochromat 100×/1.45 oil lens (KEYENCE). The software ImageMagic (v7.1.0-37) was used to standardize image sizes, ensuring consistency and resolving resolution discrepancies between microscopes. Images were subsequently processed using a Java-based program and then analyzed using CalMorph (ver 1.3). Complete dataset is provided in Supplementary File 1, Table 4.

To compare the morphology of *C. jadinii* and *S. cerevisiae*, differences in microscopy techniques [bright-field (BF) microscopy for *C. jadinii* and phase-contrast (PC) microscopy for *S. cerevisiae*] were corrected. Data normalization and parameter selection were performed to minimize bias introduced by the different microscopy setups. To address potential discrepancies, image sizes were standardized using ImageMagick software to ensure resolution consistency. To further reduce experimental bias, two strains from each species (*C. jadinii*: NBRC0987, ATCC9950; *S. cerevisiae*: BAM, BAQ) were randomly selected, and their morphological data were collected under both microscopy conditions (*n* = 5). Comparative analysis showed no significant differences in eight morphological parameters between the two microscopy setups for any strain (Supplementary Fig. 3). These morphological parameters were subsequently used for cross-species comparisons.

Additionally, we used ATCC9950 as the reference strain to detect morphological differences between each yeast strain and the reference strain. Morphological values for each trait were normalized accordingly. The morphological data (*n* = 5) were normalized by applying a one-way ANOVA using a generalized linear model (GLM), and values were then converted to Z-scores using a Wald test, as previously described (Itto-Nakama *et al*. 2021).

### High-throughput phenotyping of fitness traits

Fitness traits were assessed via high-throughput phenotyping using a microcultivation approach in biological replicates (*n* = 3) (Supplementary Table 6 in File 1). Strains were inoculated in 150 μL of YPD medium, incubated overnight at 30°C, and transferred to 96-well plates containing various media (Supplementary Table 3 in File 1). To reduce residual nutrients introduced by preculturing in YPD, we harvested and thoroughly washed the cells before inoculation. Cultivation was performed for 48 h at 30°C in a microplate reader (Thermo Scientific Varioskan LUX) with absorbance readings at 610 nm every 20 min. Growth rates were calculated using PRECOG (Fernandez-Ricaud *et al*. 2016) and normalized to standard YPD conditions, representing 1,104 data points for each of the three biological replicates.

After reducing dimension by principal component analysis (PCA), hierarchical cluster analysis (HCA) was performed using the hclust function with the average linkage method on the maximum distance matrix derived from the first 11 PCs (cumulative contribution ratio: 90%). Bootstrapping with 1,000 iterations was conducted using the pvclust package of R to assess cluster stability. The significance of each cluster was estimated from the approximately unbiased probability value (AU *P*-value) using the pvclust function as previously described (Ohnuki *et al*. 2017).

### Whole-genome sequencing analysis

Genomic DNA from 20 *C. jadinii* strains was extracted using Genomic-tip 100/G kit (QIAGEN). Sequencing libraries were prepared with the MiSeq Reagent Kit (v3) and analyzed on an Illumina MiSeq platform (2 × 300 bp).

After adapter trimming and quality filtering with fastp (v0.21.0; Chen *et al*. 2018), the paired-end reads were aligned to the *C. jadinii* reference genome ATCC9950 with BWA-MEM (v0.7.17; Li and Durbin 2009). Alignment quality was assessed by summarizing, for each strain, the number of QC-filtered read pairs, the number and percentage that mapped, the genome-wide mean coverage depth, and the mean mapping-quality score (Supplementary Table 5 in File 1). All 20 datasets showed ≥98% mapping, mean depths of ∼60–160×, and mean MapQ values of ≈57, confirming that the data are suitable for reliable variant discovery. High-quality variants were then called and hard-filtered with GATK (v4.1.8.0; McKenna *et al*. 2010), and ploidy levels were inferred from read-depth and allele-frequency profiles using CNVpytor (v1.3.1; Suvakov *et al*. 2021).

Totally 451,302 biallelic segregating sites detected among the 20 *C. jadinii* strains were used to elucidate their phylogenetic relationships. Pairwise identity by state values between strains were calculated by using SNPRelate package. Hierarchical clustering was performed on the distance matrix by using pvclust package. To assess the robustness of hierarchical clustering, the pvclust function was run with 1,000 bootstrap replications to estimate approximately unbiased (AU) *P*-values for each cluster.

### Gene prediction for ATCC9950 (NBRC0988)

Gene prediction utilized RNA-seq data (GSM8138954) aligned to the *C. jadinii* ATCC9950 (NBRC0988) genome using HISAT2 (v2.2.1) (Kim *et al*. 2015). Transcript assembly was performed with StringTie (v2.2.1) (Pertea *et al*. 2015), and coding sequences were predicted using AUGUSTUS (v3.2.1) (Stanke *et al*. 2006). Functional annotations were validated with BUSCO (v5.7.0) (Manni *et al*. 2021) and BLAST (v2.14.0).

### Discrimination analysis

Partial least squares discriminant analysis (PLS-DA) was performed using the mixOmics package of R to distinguish between *C. jadinii* and *S. cerevisiae* within both morphological traits data and fitness traits data were used as predictor variables. The PLS-DA model was fitted, generating a score matrix for discriminating between the defined classes. To identify the most influential variables contributing to the discrimination, variable importance in projection (VIP) scores were calculated using the PLSDA.VIP function.

## Results

### Genetic variation in wild *C. jadinii* strains

**Fig. 1.**
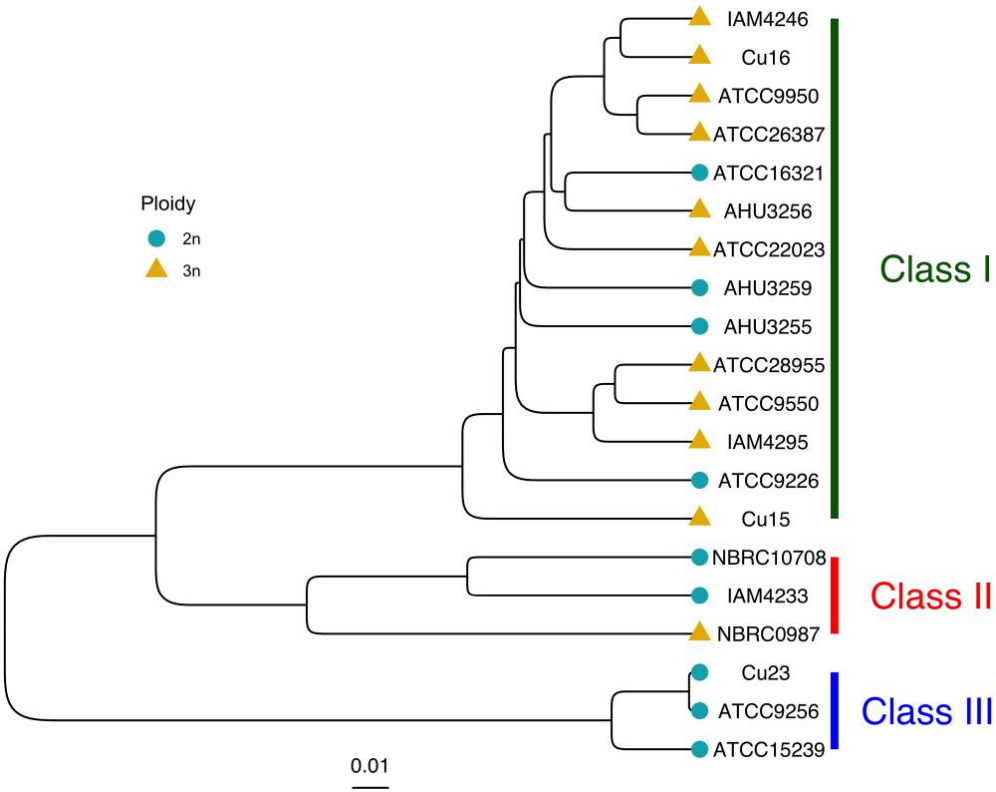
Hierarchical clustering tree of 20 wild *C. jadinii* strains based on biallelic SNPs. Light blue circle represents diploid, yellow triangle represents triploid. Three classes were identified which were named Class I (dark green), Class II (red), and Class III (blue). Scale bar represents 1% of genetic divergence. Branch of each of three cluster dendrogram stands for significant groups detected at AU *P*-values >0.95.

As a first step toward investigating genetic diversity and broadly genotype-phenotype relationships among wild *C. jadinii* strains, a hierarchical clustering tree (average linkage) was constructed based on biallelic SNPs (Fig. 1). The analysis identified three clades, designated as Class I (dark green), Class II (red), and Class III (blue), which reflect genetic divergence and represent three distinct genetic groups. Ploidy levels for each strain were determined using SNP allele-frequency ratios, as described in “Materials and methods.” Diploid (2n) strains were identified by a 1:1 SNP ratio, while triploid (3n) strains exhibited a 1:2 ratio. Aneuploidy was defined as deviations from these expected ratios and further confirmed by variations in coverage depth, which reflect relative chromosome copy number. Strains with stable SNP ratios and proportional coverage depths were categorized as euploid, while those with detectable deviations were categorized as aneuploid.

Integration of ploidy data with phylogenetic analysis revealed distinct patterns among the clades. Class I and Class II contained both diploid and triploid strains, whereas Class III consisted exclusively of diploid strains. Aneuploidy was predominantly observed in triploid strains, while all diploid strains exhibited euploid chromosome configurations (Supplementary Table 1 in File 1). Statistical analysis supported this observation: a chi-square test revealed a significant association between ploidy (2n vs 3n) and aneuploidy (χ^2^ = 4.27, *P* = 0.0030), indicating that triploid strains are more prone to aneuploidy than diploid strains. This finding aligns with previous studies suggesting that triploidy increases chromosomal instability, often leading to aneuploidy (Pfau and Amon 2012). Overall, these results illustrate the genetic diversity and variation in ploidy within *C. jadinii*, providing a foundation for further studies on genotype–phenotype relationships in this species.

### Morphological variation in wild *C. jadinii* strains

To assess morphological diversity among 20 wild *C. jadinii* strains, we analyzed 31 morphological traits related to cell size, shape, and other cellular characteristics using CalMorph-PC(10) (Itto-Nakama *et al*. 2023). PCA was performed on all traits to visualize and interpret morphological variation across the strains. The first two principal components (PC1 and PC2) accounted for 55.29 and 20.07% of the total variance, respectively, with a cumulative contribution of 75.36% (Supplementary Fig. 1), indicating that most morphological variation was captured by these two components.

**Fig. 2.**
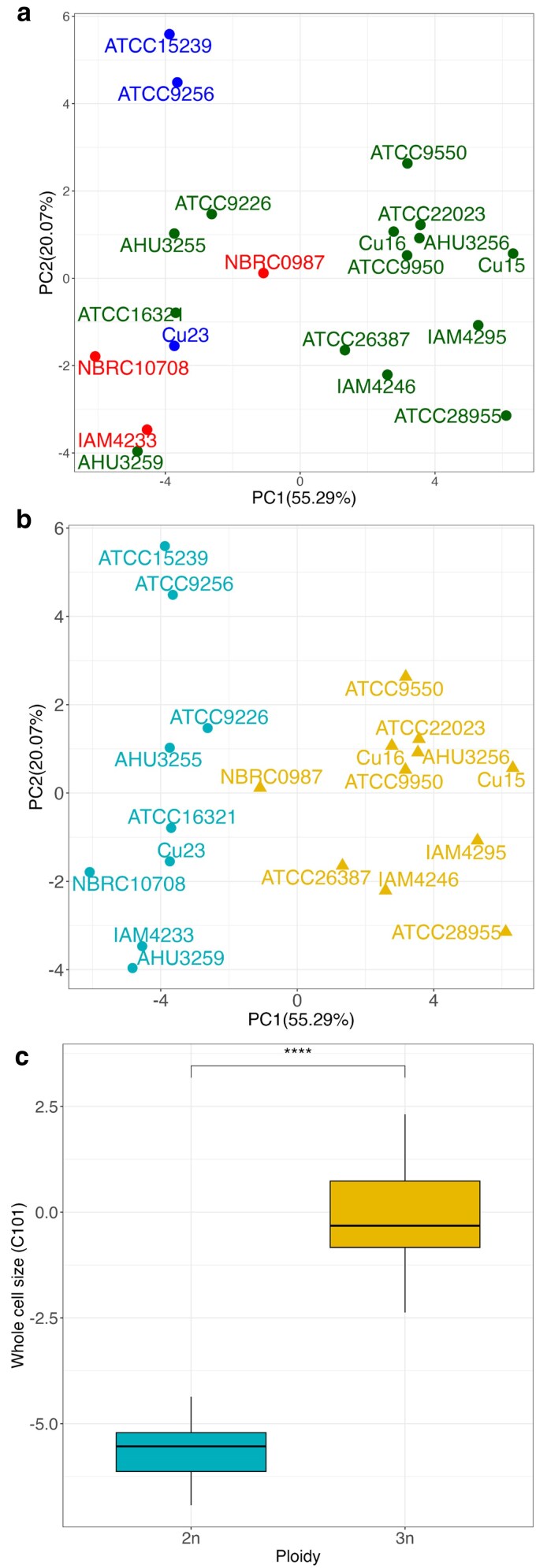
Morphology variation of *C. jadinii*. a) Morphology distribution of *C. jadinii* using PCA. Color used in the legends is the same as genetic classification in Fig. 1. Percentages in parentheses on each axis present the contribution ratio. b) Morphology distribution of *C. jadinii*. The color legends of each strain are the same as different ploidy used in Fig. 1; blue circle denotes triploid and yellow triangle denotes diploid. Percentages in parentheses on each axis present the contribution ratio. c) Difference in Z-values for whole cell size between diploid and triploid strains. Asterisks (****) indicate *P* < 0.0001 by Wilcoxon–Mann–Whitney test (*n* = 9, 11).

Interestingly, morphological phenotypes did not cluster according to genetic clades (Fig. 2a), suggesting that morphological variation is largely independent of genetic lineage. Instead, morphology varied significantly by ploidy level (Fig. 2b). Diploid (light blue circle) and triploid (yellow triangle) strains were clearly separated in the 2D morphological space, with triploid strains showing higher PC1 scores. This finding suggests that ploidy level plays a critical role in shaping phenotypic traits in *C. jadinii*.

One of the most striking differences between diploid and triploid strains was cell size (Fig. 2c). Triploid strains exhibited significantly larger cell sizes than diploid strains (Wilcoxon–Mann–Whitney test, *P* = 1.19 × 10^−5^, *n* = 9 for diploids and *n* = 11 for triploids). Thus, morphological variation among wild *C. jadinii* strains is strongly associated with ploidy level rather than genetic lineage. This observation is consistent with findings in other yeast species, such as *S. cerevisiae* (Fukuda 2023) and *S. pombe* (Patterson *et al*. 2021).

### Variation in fitness traits of wild *C. jadinii* strains

To investigate diversity in fitness traits, we performed high-throughput growth phenotyping under 24 distinct conditions, including various carbon sources, toxins, and environmental stressors (Supplementary Table 3 in File 1). Fitness was quantified as specific growth rates normalized to performance in standard YPD medium, with measurements recorded over a 48-h period. Thus, fitness data from 23 conditions (excluding YPD) were used for subsequent analysis (Supplementary Table 6 in File 1).

**Fig. 3.**
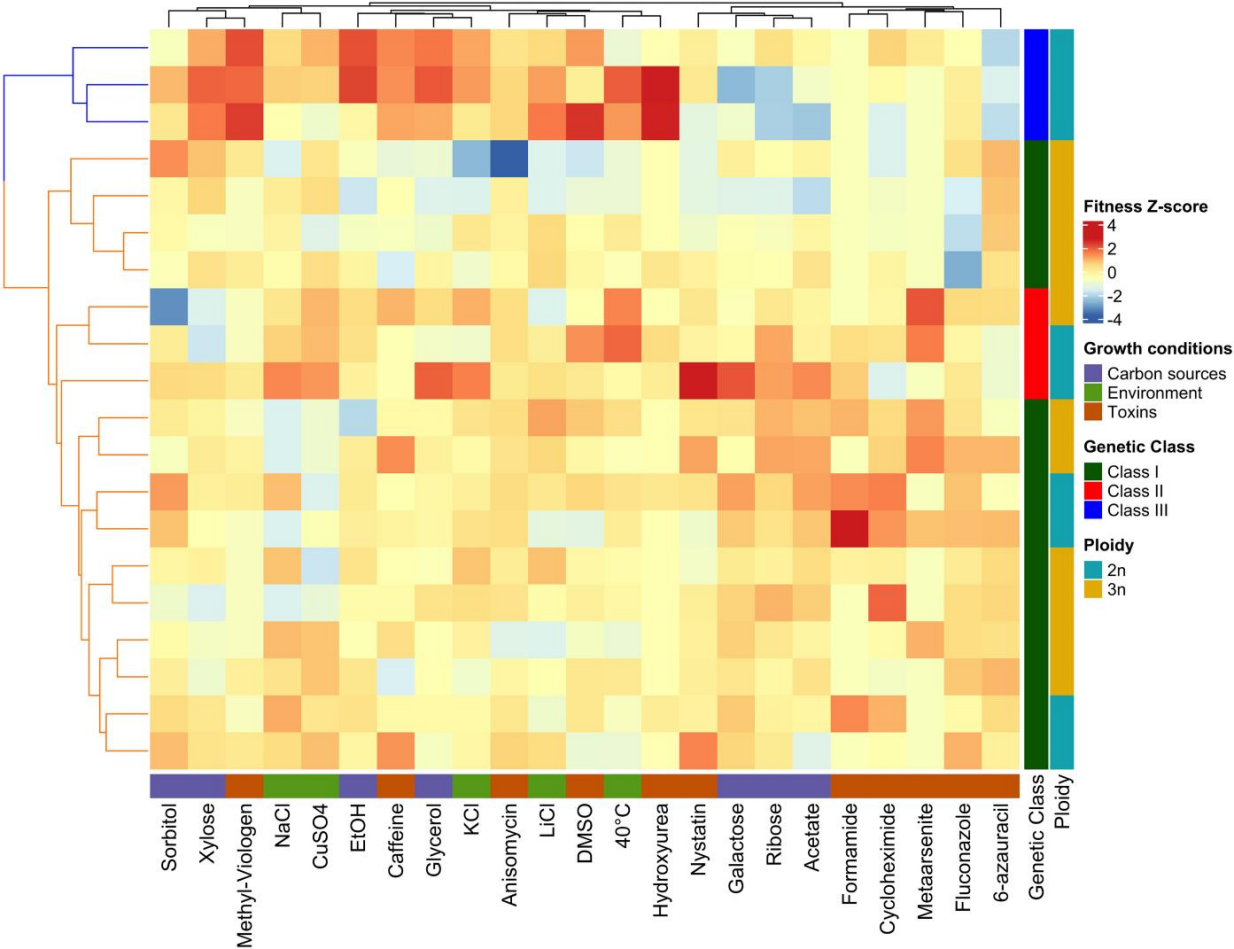
Heat map of fitness traits in 20 *C. jadinii* strains. Fitness traits were normalized for each condition to YPD (yeast extract 10 g/L, polypeptone 20 g/L, dextrose 20 g/L). The high growth rates are depicted in red and slow growth rates in blue in the color scale. Cluster dendrogram at the left side of the heatmap was constructed from average linkages of maximum distance matrix calculated from PC scores of 11 PCs (CCR = 90%) from PCA of 23 traits. Blue and orange branches in cluster dendrogram stand for significant groups detected at AU *P*-values >0.95. The bar at the bottom of the heatmap indicates the type of growth condition (purple = “Carbon sources,” green = “Environment”, orange = “Toxins”). Bars on the right indicate genetic class: “Class I” in dark green, “Class II” in red, “Class III” in blue: and ploidy (2n in yellow, 3n in teal).

This analysis revealed substantial variation in growth rates among the 20 strains across the tested conditions. A heatmap (Fig. 3) visualized the normalized growth rates, with higher values shown in red and lower values in blue. PCA of 23 fitness traits identified 11 principal components that together explained 90% of the total variance (Supplementary Fig. 2). Hierarchical clustering revealed two major phenotypic clusters (Fig. 3, left panel), represented by blue and orange branches with strong statistical support (AU *P*-value > 0.95). Additionally, clustering was performed on both the rows (strains) and the columns (fitness traits), as clarified in the figure legend. No strong separation of conditions into distinct functional groups (e.g. carbon sources vs stressors) was observed; instead, most conditions formed a more diffuse pattern, suggesting that phenotypic responses may involve overlapping or multifactorial mechanisms. Ploidy status (2n vs 3n) in the heatmap did not reveal a clear correlation with the major phenotypic clusters. Comparisons of 2n vs 3n strains under each condition (Supplementary Fig. 4) indicate that only three of the fitness traits displayed notable differences based on ploidy, suggesting that overall fitness differences are not strictly dependent on ploidy level.

The blue cluster predominantly consisted of Class III strains, which exhibited significantly different fitness profiles compared to Class I (green) and Class II (red) strains. The distinct fitness traits of Class III strains suggest unique adaptive mechanisms, consistent with their observed genetic divergence. Notably, although Class III strains were exclusively diploid, their elevated growth rates were not observed across diploid strains in other classes according to the result of PCA with combination data of all diploid strains (Supplementary Fig. 5), indicating that the superior performance of some fitness traits in Class III is not attributable to ploidy status alone but instead reflects lineage-specific genetic factors.

### Distribution of homozygous single-nucleotide polymorphisms and relationship with fitness traits

**Fig. 4.**
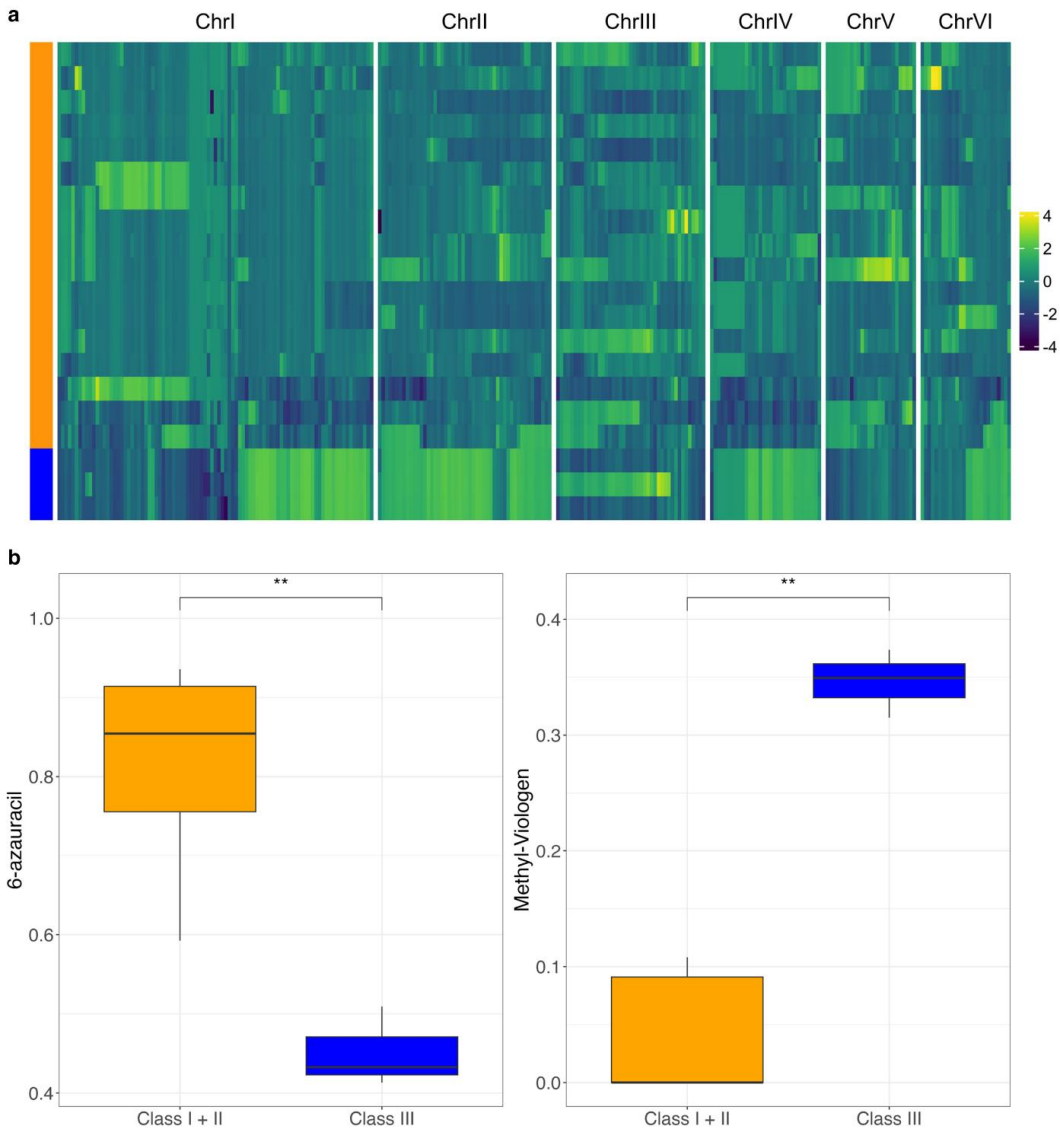
Relationship between homozygous SNPs distribution on fitness traits. a) Homozygous SNPs distribution on genome-wide scale. Heatmap showing the scaled homozygous SNP density across 50,000 bp genomic windows for 20 strains. Rows represent strains, grouped into “Class I + II” (orange) and “Class III” (blue) based on the result of cluster dendrogram presented in Fig. 3. Columns represent continuous genomic windows, with color indicating the relative density of homozygous SNPs, standardized across windows. Chromosomes are represented in separate column splits, and the figure highlights regions with varying SNP densities across the genome. b) Fitness differences between “Class I + II” strains and “Class III” strains. 6-azauracil (left) and Methyl-Viologen (right). Asterisks (**) indicate *P* < 0.01 by Wilcoxon–Mann–Whitney test (*n* = 17, 3) for both conditions.

Genomic variation, particularly in homozygous single-nucleotide polymorphisms (SNPs), often reflects adaptive processes and functional divergence, potentially underlying phenotypic differences. To determine whether such patterns exist in *C. jadinii*, we analyzed the genome-wide distribution of homozygous SNPs across the 20 strains. The distribution was visualized using a heatmap with 50,000 bp genomic windows (Fig. 4a).

Strains were categorized into “Class I + II” (orange) and “Class III” (blue), based on hierarchical clustering results shown in Fig. 3. Distinct patterns emerged, with Class III strains exhibiting increased homozygous SNP density in specific chromosomal regions, particularly on the right arms of ChrI and ChrVI, and large portions of ChrII and ChrIV. These findings suggest that unique genomic events or selective pressures may have influenced the evolutionary trajectory of Class III strains.

Phenotypic analysis revealed significant differences in fitness under stress conditions, particularly in response to 6-azauracil and methyl viologen. Class I + II strains exhibited significantly higher fitness under 6-azauracil treatment than Class III strains (Fig. 4b, *P* = 0.0018), whereas Class III strains showed superior fitness under methyl viologen stress (Fig. 4b, *P* = 0.0047 by Wilcoxon–Mann–Whitney test). These results highlight a correlation between genomic differences in homozygous SNP distribution and phenotypic divergence in fitness traits among *C. jadinii* strains.

### Genomic and fitness differences between *C. jadinii* and *S. cerevisiae*

**Fig. 5.**
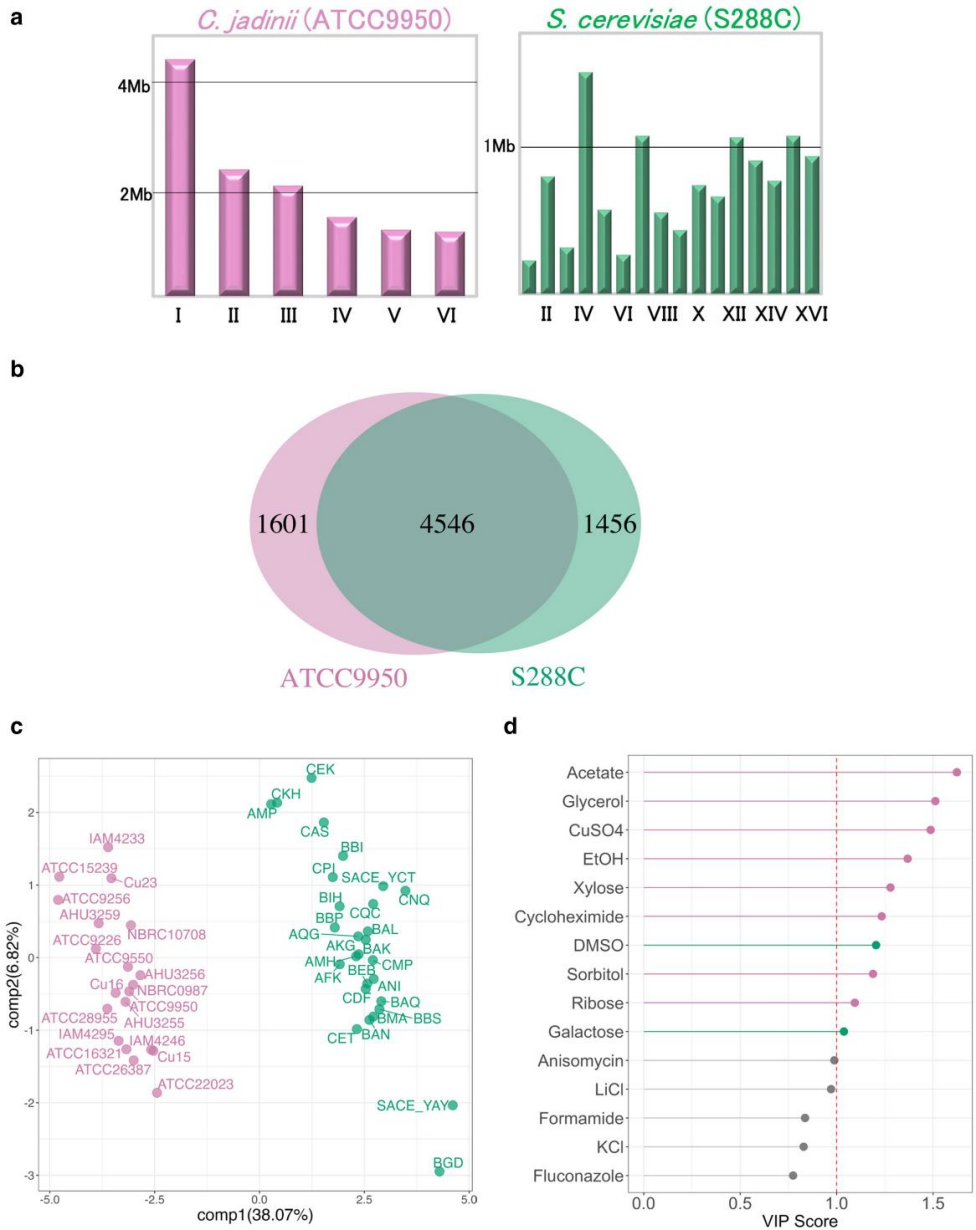
Fitness differences between *C. jadinii* and *S. cerevisiae*. a) Chromosomal distribution of *C. jadinii* and *S. cerevisiae* based on chromosome length. Chromosomes of *C. jadinii* (pink) and *S. cerevisiae* (green) are represented according to their respective sizes. The y-axis indicates chromosome length in megabases (Mb). b) Homologous and unique genes between *C. jadinii* (ATCC9950) and *S. cerevisiae* (S288C) based on gene prediction results for *C. jadinii* compared with the Saccharomyces Genome Database (SGD) using BLAST analysis. The overlapping region indicates 4,546 homologous genes (E-value < 1e−10), while 1,601 and 1,456 genes are unique to *C. jadinii* and *S. cerevisiae*, respectively. c) Distribution of *C. jadinii* and *S. cerevisiae* based on fitness using PLS-DA. Same color is used in the distribution of *C. jadinii* (pink) and *S. cerevisiae* (green) based on their fitness data, respectively. The percentages in parentheses along each axis represent the contribution ratios of each component to the model. d) Variable importance in projection (VIP) scores for fitness data of *S. cerevisiae* and *C. jadinii*. Fifteen VIP scores derived from the PLS-DA model are plotted, highlighting the most influential variables distinguishing between *S. cerevisiae* and *C. jadinii*. The bars are color-coded based on the yeast species, with green representing variables where *S. cerevisiae* scored higher and pink indicating those where *C. jadinii* scored higher. A gray color is used for variables that did not show a clear dominance. The red dashed line represents the threshold value of 1, above which variables are considered to have significant importance in the discrimination between the two classes.

To gain a broader perspective on the genetic and phenotypic diversity of *C. jadinii*, we conducted a comparative analysis with the model yeast *S. cerevisiae*. Regarding genome organization, *C. jadinii* has six chromosomes ranging in size from approximately 1.2–4 Mb, with a total genome length of 13.12 Mb (Yasukawa *et al*. 2022), whereas *S. cerevisiae* has 16 chromosomes ranging from 0.23 to 1.53 Mb, with a total genome length of 12.07 Mb (Fig. 5a). Comparative genomic analysis using BLAST revealed that *C. jadinii* (ATCC9950) shares 4,546 homologous genes with *S. cerevisiae* (S288C) but also harbors 1,601 unique genes not found in *S. cerevisiae*. Conversely, *S. cerevisiae* contains 1,456 unique genes absent in *C. jadinii* (Fig. 5b). These findings emphasize genetic differences between these species, reflecting divergences in genome organization and potential functional roles.

To compare fitness traits, we analyzed growth under diverse environmental and chemical conditions using partial least squares discriminant analysis (PLS-DA), a supervised classification method combining partial least squares (PLS) regression with discriminant analysis. The analysis revealed a clear distinction between the two species, with the first two components accounting for 38.07 and 26.82% of the variance, respectively (Fig. 5c). This separation underscores significant differences in metabolic capacity and stress-response mechanisms.

Variable importance in projection (VIP) scores identified key factors driving this separation (Fig. 5d). Notably, *C. jadinii* exhibited superior fitness under conditions utilizing acetate, glycerol, ethanol (EtOH), and xylose as carbon sources, reflecting its broad metabolic versatility. Additionally, its enhanced growth in the presence of CuSO_4_ suggests a remarkable tolerance to heavy metal stress. These traits highlight *C. jadinii*'s potential advantages in fermentation processes using unconventional substrates or extreme environments.

### Functional differentiation of *FPS1* between *C. jadinii* and *S. cerevisiae*

**Fig. 6.**
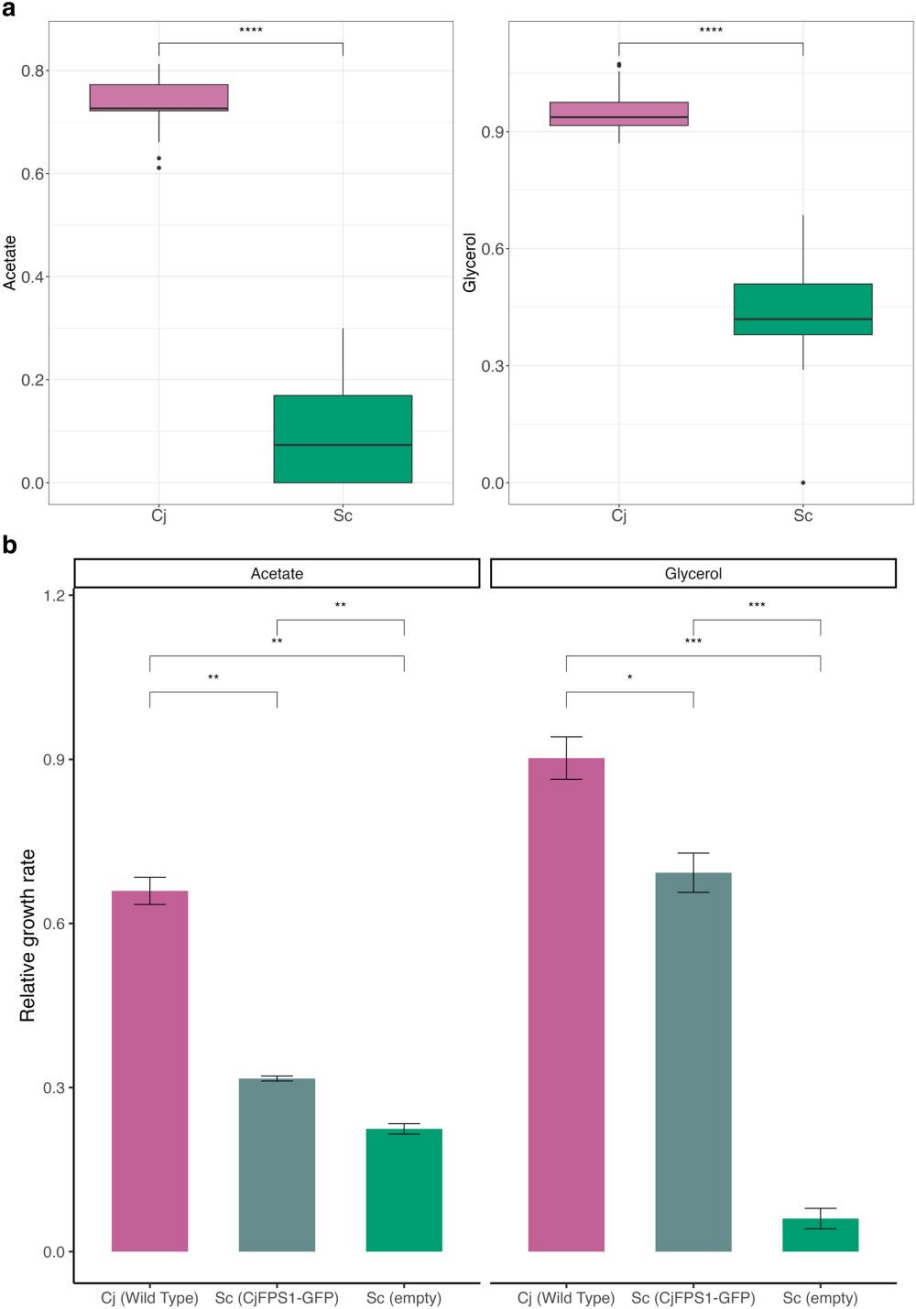
Growth performance of *S. cerevisiae* BY4741 expressing *CjFPS1*. a) Box plot shows the fitness of *C. jadinii* and *S. cerevisiae* in media containing acetate and glycerol as the sole carbon sources. Asterisks indicate significance levels determined by Wilcoxon–Mann–Whitney test (*n* = 20, 28): **** for *P* < 0.0001. b) Bar plot shows the relative growth rate of the three strains cultivated with either acetate or glycerol as carbon source: *C. jadinii* wild-type strain ATCC9950 (pink, *n* = 3), *S. cerevisiae* BY4741 expressing CjFPS1-GFP (teal, *n* = 3), and BY4741 carrying the empty CEN vector (green, *n* = 3); horizontal brackets denote Welch's *t*-tests for each carbon source, with error bar stands for standard deviation and significance indicated as *P* < 0.05 (*), *P* < 0.01 (**), *P* < 0.001 (***).

As illustrated in Figs. 5d and 6a, *C. jadinii* demonstrated enhanced fitness in acetate and glycerol-based media. Given that the *S. cerevisiae FPS1* gene encodes a plasma membrane channel involved in glycerol and acetate metabolism (Tamás *et al*. 1999; Vilela-Moura *et al*. 2011), we hypothesized that the homologous gene in *C. jadinii* (*CjFPS1*) contributes to its superior fitness under these conditions.

To test this hypothesis, we cloned *CjFPS1* and expressed it in the *S. cerevisiae* wild-type strain BY4741 as a CjFPS1-GFP fusion protein. Growth was then compared with (i) the BY4741 empty-vector control and (ii) the *C. jadinii* wild-type reference strain ATCC9950 (Supplementary Table 7 in File 1). As shown in Fig. 6b, CjFPS1-GFP expression enhanced growth rates in both acetate and glycerol media. In acetate medium, the CjFPS1-GFP strain grew 1.41-fold faster than the empty-vector control (Welch's *t*-test, *P* = 0.0038) and reached ∼48% of the ATCC9950 growth rate. In glycerol medium, the effect was even stronger: heterologous CjFPS1 yielded an 11.46-fold increase relative to the control (*P* = 5.50 × 10^−4^) and recovered ∼77% of the *C. jadinii* rate.

These results confirm that CjFPS1 confers a substantial advantage for *S. cerevisiae* growth on nonfermentable carbon sources, although further characterization is required to confirm the underlying mechanisms fully. Similar results have also been reported previously, indicating that glycerol facilitators from various yeast species, including *C. jadinii*, can significantly improve glycerol utilization in *S. cerevisiae* (Klein *et al*. 2016).

### Morphological differences between *C. jadinii* and *S. cerevisiae*

**Fig. 7.**
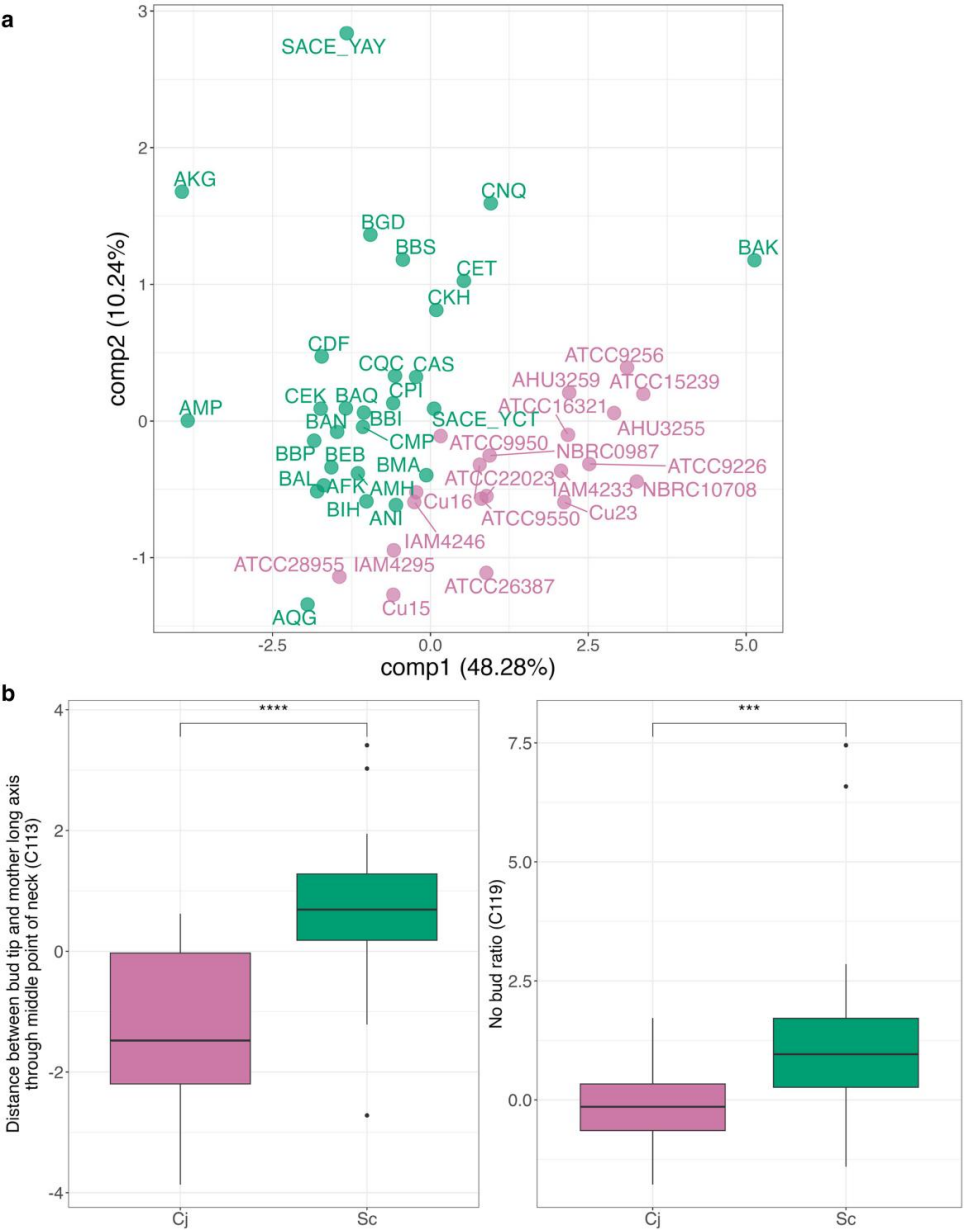
Morphological differences between *C. jadinii* and *S. cerevisiae*. a) Distribution of *C. jadinii* and *S. cerevisiae* based on morphology using partial least squares discriminant analysis (PLS-DA). Green and pink indicates *S. cerevisiae* and *C. jadinii*, respectively. The percentages in parentheses along each axis represent the contribution ratios of each component to the model. b) Comparison of two representative morphological parameters between *C. jadinii* and *S. cerevisiae*. The left panel shows the Z-value of “Distance between Bud Tip and Mother Long Axis through the Middle Point of the Neck,” the right panel depicts the Z-value of “No Bud Ratio.” Asterisks denote significance levels determined by the Wilcoxon–Mann–Whitney test (*n* = 20, 28): *** for *P* < 0.001 and **** for *P* < 0.0001.

Morphological traits of *C. jadinii* and *S. cerevisiae* were compared using CalMorph-PC(10). PLS-DA revealed a clear morphological distinction between the two species, with the first two components explaining 48.28 and 10.24% of the variance, respectively (Fig. 7a).

Key morphological parameters driving this separation are highlighted in Fig. 7b. Notably, *C. jadinii* exhibited a significantly lower “No Bud Ratio” than *S. cerevisiae* (Wilcoxon–Mann–Whitney test, *P* = 2.84 × 10^−4^). Additionally, “Distances between Bud Tip and Mother Long Axis through the Middle Point of the Neck” was markedly lower in *C. jadinii* (Wilcoxon–Mann–Whitney test, *P* = 2.66 × 10^−6^). These results highlight distinct morphological differences between the two yeast species, reflecting variations in cellular architecture and growth dynamics.

### Relationship between genetic diversity and trait profile variation

Genetic diversity was investigated using genome information from the *C. jadinii* and *S. cerevisiae* populations analyzed in this study. Nucleotide diversity (*π*) for *C. jadinii* was calculated as 18 × 10^−3^, approximately four times higher than that of *S. cerevisiae* (4 × 10^−3^). This finding highlights the notably high genetic diversity within *C. jadinii*, despite the limited number of strains analyzed. The observed genetic diversity in *C. jadinii* may reflect historical population dynamics or adaptation to diverse ecological niches.

**Fig. 8.**
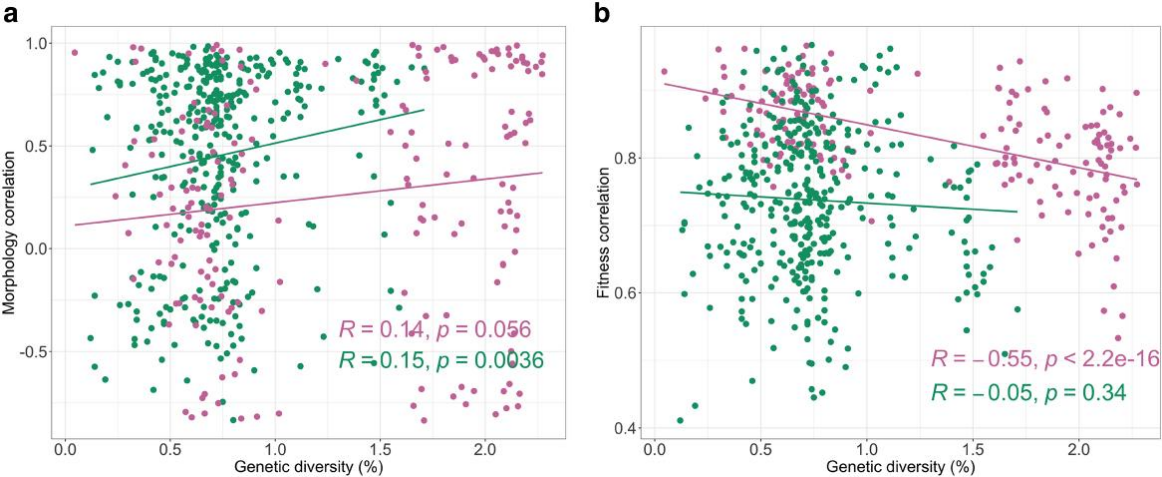
Correlation between genetic diversity and trait profile variation. Pairwise genetic diversity is plotted against pairwise Pearson correlation coefficients for morphology traits (a) and fitness traits (b), displaying with Pearson correlation coefficients and *P*-value. The same color in Fig. 6 is used, green stands for *S. cerevisiae* and pink indicates *C. jadinii*.

To examine the relationship between genetic diversity and phenotypic variation, we analyzed correlations between genetic similarity and trait profiles. For morphological traits (Fig. 8a), a weak but statistically significant positive correlation was observed in *S. cerevisiae* (R = 0.15, *P* = 0.0036), suggesting a slight association between genetic similarity and morphological resemblance. In contrast, *C. jadinii* showed no significant correlation (*R* = 0.14, *P* = 0.056), indicating that genetic variation has minimal influence on morphological traits in this species.

For fitness traits (Fig. 8b), a strong negative correlation was observed in *C. jadinii* (*R* = −0.55, *P* = 2.2 × 10^−16^), suggesting that greater genetic diversity corresponds to increased phenotypic variability. Conversely, no significant relationship was detected in *S. cerevisiae* (*R* = −0.05, *P* = 0.34). These results indicate that fitness traits in *C. jadinii* are more strongly influenced by genetic differences, potentially reflecting diverse adaptive strategies or responses to ecological challenges.

Overall, these findings demonstrate that genetic diversity has a more pronounced impact on fitness traits in *C. jadinii* than in *S. cerevisiae*. The weak or absent correlations for morphological traits suggest that morphological variation is less influenced by genome-wide genetic diversity and may instead be governed by other factors such as ploidy variation or uncharacterized regulatory mechanisms.

## Discussion

This study comprehensively examined the genomic characteristics, morphological diversity, and fitness traits of *C. jadinii* using whole-genome sequencing data, large-scale analysis of morphological features, and fitness assays under various conditions. Our findings provide a more detailed understanding of *C. jadinii*'s genetic diversity and adaptive capacity than previously available.

### Ploidy and morphological variation

Ploidy had a stronger influence on cell morphology than lineage-specific genetic factors in *C. jadinii*. Triploid strains exhibited significantly larger cell sizes than diploid strains. A similar trend has been observed in *S. cerevisiae* and *S. pombe*, where polyploidization affects cell cycle regulation and overall growth (Patterson *et al*. 2021; Fukuda 2023). These findings suggest that changes in chromosome copy number represent a widespread strategy for morphological variation in yeast species.

### Lineage-specific genetic factors and fitness traits

In contrast, fitness traits-including stress tolerance, utilization of nonconventional carbon sources, and toxin resistance were primarily governed by lineage-specific genetic factors. This study found no evidence that ploidy (diploid vs triploid) strongly influenced fitness traits. Instead, analysis of homozygous SNP distribution suggested that lineage-specific genetic variations play a key role. Notably, Class III strains, which exhibited distinct stress responses (e.g. differential resistance to 6-azauracil and methyl viologen), harbored “hotspots” of homozygous SNP accumulation. These regions may reflect adaptation to specific ecological or evolutionary pressures. Future studies integrating SNP analysis with multiomics approaches and investigating *C. jadinii* strains from diverse ecological and industrial environments will provide deeper insights into the evolution of these adaptive traits.

### Comparative genomics of *C. jadinii* and *S. cerevisiae*

Comparative analyses between *C. jadinii* and *S. cerevisiae* revealed notable differences in chromosomal structure, gene content, and phenotypic profiles. While *C. jadinii* has only six chromosomes, its genome is comparable in size to that of *S. cerevisiae*. Importantly, *C. jadinii* retains numerous genes absent in *S. cerevisiae*, which may explain its ability to grow on nonconventional substrates such as acetate, xylose, and glycerol, as well as its high tolerance to heavy metals like copper. Consistent with this view, *C. jadinii* also out-performed largely derived *S. cerevisiae* panel under ethanol stress, echoing a recent report of superior ethanol tolerance in *C. jadinii* (Vieira-Lara *et al*. 2024). Direct benchmarking against ethanol-tolerant industrial yeasts will be an informative next step.

Functional evidence further supports a genetic basis for some traits: *CjFPS1* homolog demonstrated that expressing *CjFPS1* in *S. cerevisiae* enhanced growth on glycerol and acetate. This suggests that *C. jadinii*'s ability to utilize nonconventional substrates may depend on specific transporters and metabolic genes. However, while these results suggest a role for *CjFPS1* in facilitating growth on nonfermentable carbon sources, further investigation including controls such as expressing *ScFPS1* under the same promoter or expressing *CjFPS1* from its native locus, are necessary to conclusively validate the specific contribution of *CjFPS1*.

In addition, although performing GO enrichment analysis on genes unique to *C. jadinii* and *S. cerevisiae* could provide deeper understanding into functional divergence, the current limitations in comprehensive and well-curated function annotations for *C. jadinii* restrict the interpretability and statistical robustness of such analyses. Improving functional prediction of *C. jadinii* genes represents an important area for future research, which will enhance our understanding of its genetic and metabolic capabilities for industrial applications and microbial fermentation optimization.

### Morphological distinctions of *C. jadinii*

*Cyberlindnera jadinii* also exhibited distinct morphological traits compared to *S. cerevisiae*. For example, *C. jadinii* had a lower “No Bud Ratio” (C119), suggesting a higher budding frequency, consistent with its high growth rate. This trait may be advantageous in industrial fermentation processes requiring high cell density cultures or short production cycles (Imura *et al*. 2020).

### Genetic diversity and its relationship with phenotypic variation

*Cyberlindnera jadinii* was found to be approximately four times more genetically diverse than *S. cerevisiae*. Statistical analyses revealed a strong negative correlation (*R* = −0.55, *P* = 2.2 × 10^−16^) between genetic similarity and differences in fitness traits, indicating that strains with greater genomic divergence showed more pronounced differences in growth performance and stress resistance. In contrast, morphological variation was not strongly associated with genetic diversity in either species. A similar trend has been observed in *Lachancea kluyveri*, where growth-related phenotypes correlate strongly with genetic distance (Jung *et al*. 2016). However, this study did not account for the potential effects of transposons or structural variations, leaving their roles in adaptation unresolved.

## Conclusion

This study elucidated how ploidy variation and genetic lineage shape the morphological and fitness traits of *C. jadinii*. Our findings highlight the species’ genetic diversity, metabolic flexibility, and stress tolerance, emphasizing its potential for industrial and biotechnological applications.

## Supplementary Material

jkaf145_Supplementary_Data

## Data Availability

Whole-genome sequencing (WGS) data for 20 samples of *C. jadinii* generated in this study have been deposited in the NCBI Sequence Read Archive (SRA) under BioProject accession number PRJNA1244612. WGS data for the reference strain ATCC9950 (NBRC0988) were obtained from a previously published dataset available under BioProject accession number PRJDB11630. Morphological data of 31 traits for all strains is listed in Supplementary Table 4 in File 1. Supplemental material available at G3 online.
