# Supplementary material for: Genetic and phenotypic insights into Cyberlindnera jadinii as a promising yeast for industrial biotechnology
Source: G3 (Bethesda). 2025 Jun 30;15(9):jkaf145. doi: 10.1093/g3journal/jkaf145 (PMC12405887; doi:10.1093/g3journal/jkaf145)
Supplement: jkaf145_Supplementary_Data [file jkaf145_supplementary_data.zip › Figure_S1_G3-2025-405956.pdf]

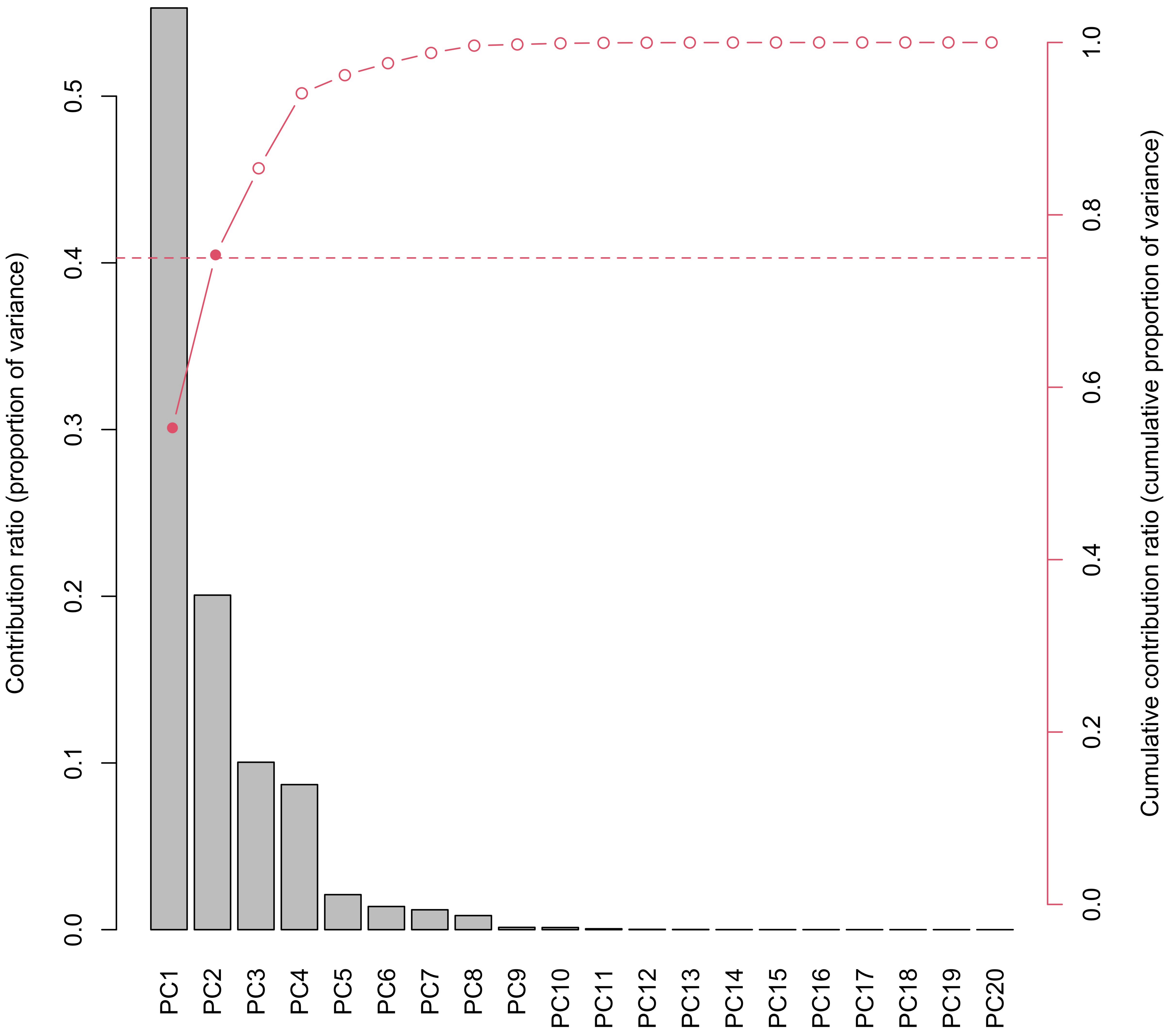

Contribution ratio (proportion of variance)
0.0
0.1
0.2
0.3
0.4
0.5
Cumulative contribution ratio (cumulative proportion of variance)
0.0
0.2
0.4
0.6
0.8
1.0
PC1
PC2
PC3
PC4
PC5
PC6
PC7
PC8
PC9
PC10
PC11
PC12
PC13
PC14
PC15
PC16
PC17
PC18
PC19
PC20
