# Supplementary material for: Genetic and phenotypic insights into Cyberlindnera jadinii as a promising yeast for industrial biotechnology
Source: G3 (Bethesda). 2025 Jun 30;15(9):jkaf145. doi: 10.1093/g3journal/jkaf145 (PMC12405887; doi:10.1093/g3journal/jkaf145)
Supplement: jkaf145_Supplementary_Data [file jkaf145_supplementary_data.zip › Supplemental_Figure_Legends_G3-2025-405956.docx]

Figure S1. Distribution of the cumulative contribution ratio of the PCA on the Z-values of the morphology traits of *C. jadinii*. The black bars represent the variance contribution ratio of each principal component (left axis), while the red curve shows the cumulative contribution ratio (right axis). The red dashed line indicates the 0.75 threshold, and the red circles mark the cumulative contribution of the first two principal components, reaching 75.36%.

Figure S2. Distribution of the cumulative contribution ratio of the PCA on the fitness traits of *C. jadinii*. The black bars represent the variance contribution ratio of each principal component (left axis), while the red curve shows the cumulative contribution ratio (right axis). The red dashed line indicates the 0.9 threshold, and the red circles mark the cumulative contribution of the first 11 principal components, reaching 90.07%.

Figure S3. Selection of non-significant parameters from CalMorph-PC and CalMorph-BF data across three randomly selected strains (NBRC0987, BAM, BAQ). Data were collected under PC and BF conditions (n=5 per condition). Parameters without significant differences were identified for each strain using the Wilcoxon-Mann-Whitney test with Benjamini-Hochberg correction. The intersection highlights 8 shared parameters that showed no significant differences across all three strains in both environments.

Figure S4. Ploidy dependent fitness comparison across 23 conditions. For each condition, box plots show the distribution of Z-normalized growth rates for diploid (2n, green) and triploid (3n, pink) strains. Horizontal brackets indicate the outcome of a Wilcoxon-Mann-Whitney test, significance levels are denoted as *P* < 0.05 (*), *P* < 0.01 (**), and “ns” for non-significant differences.

Figure S5. Principal component analysis of fitness profiles among diploid strains. Scatter plot of PC1 (39.1 % variance) versus PC2 (18.1 %) calculated from the 23 normalized fitness traits, showing diploid Class III strains (blue) clearly separated from diploid strains in Classes I + II (orange).
