# Supplementary figures and images for: Genetic and phenotypic insights into Cyberlindnera jadinii as a promising yeast for industrial biotechnology
Source: G3 (Bethesda). 2025 Jun 30;15(9):jkaf145. doi: 10.1093/g3journal/jkaf145 (PMC12405887; doi:10.1093/g3journal/jkaf145)

NBRC0987  
(15)

BAM  
(18)

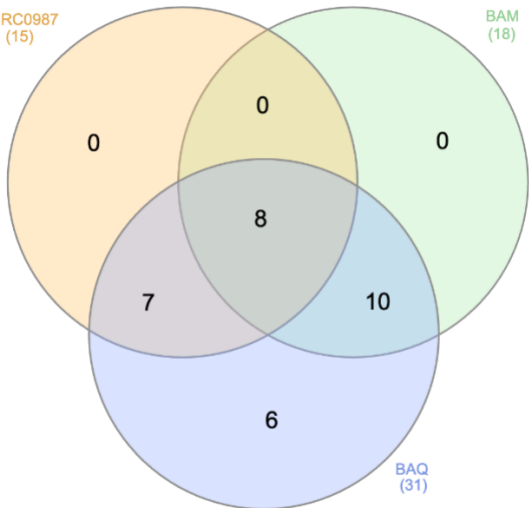

BAQ  
(31)

Supplement: jkaf145_Supplementary_Data [file jkaf145_supplementary_data.zip › Figure_S3_G3-2025-405956.pdf]

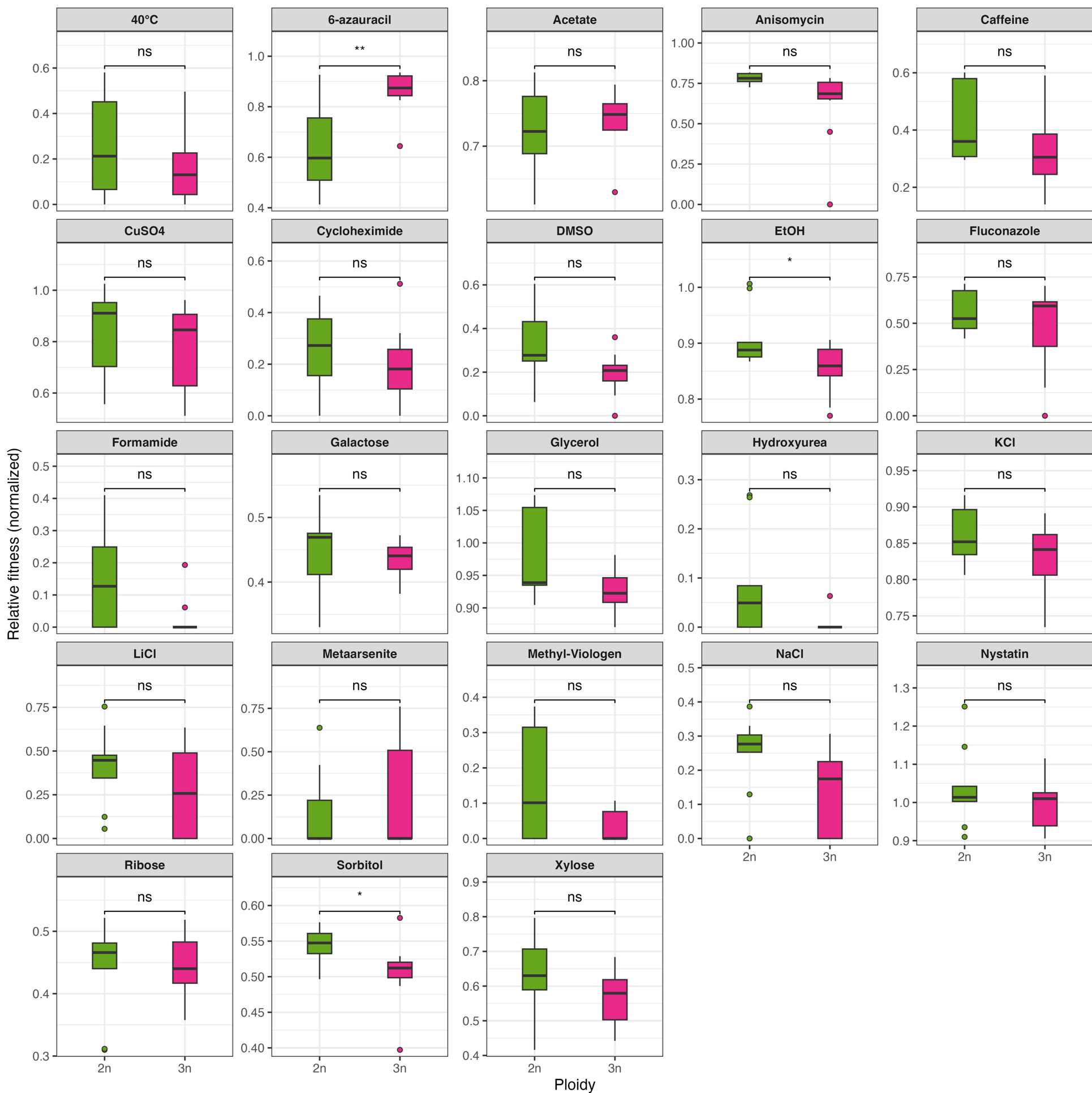

Supplement: jkaf145_Supplementary_Data [file jkaf145_supplementary_data.zip › Figure_S4_G3-2025-405956.pdf]

● ClassIII-2n ● Other Class-2n

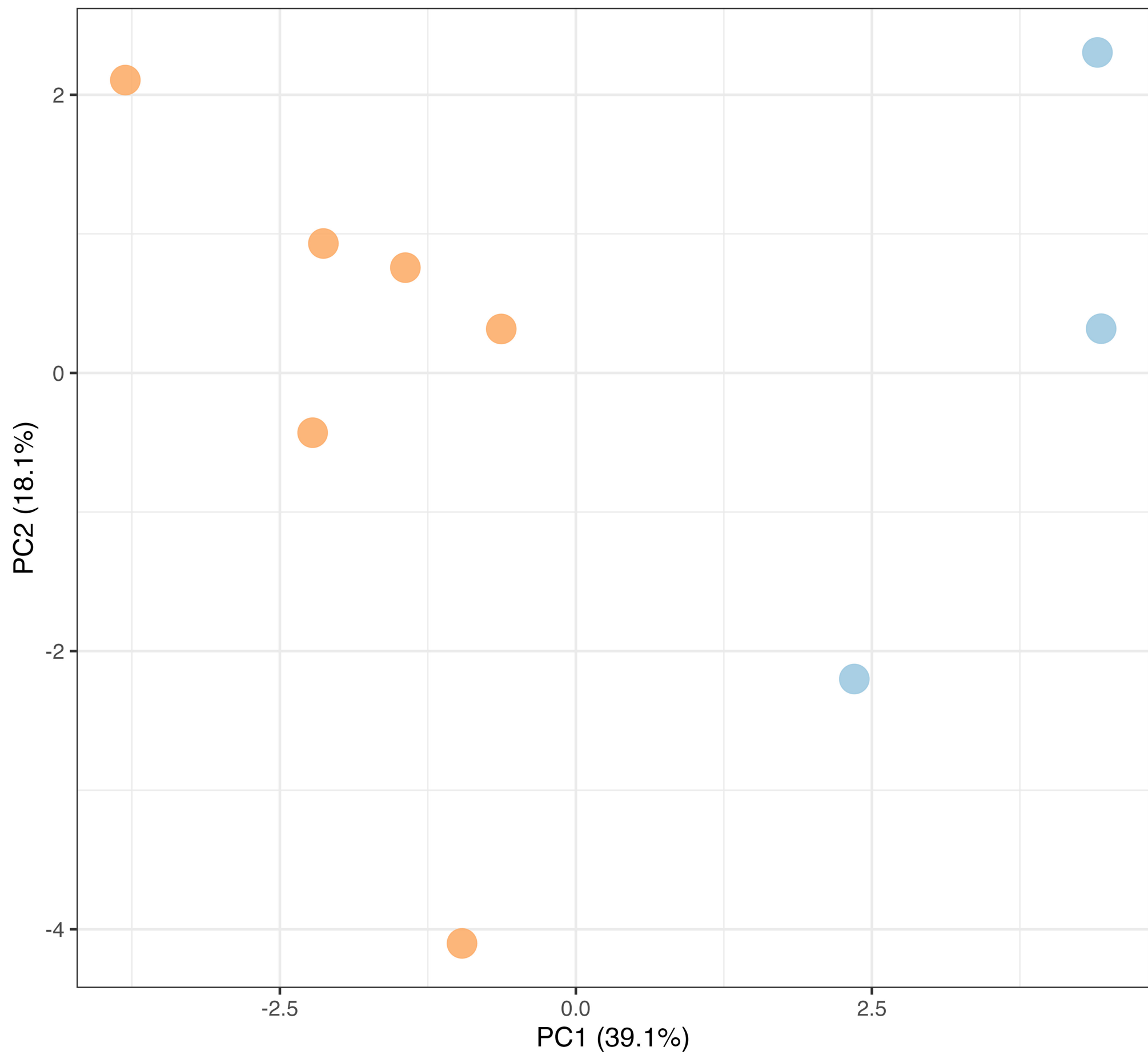

Supplement: jkaf145_Supplementary_Data [file jkaf145_supplementary_data.zip › Figure_S5_G3-2025-405956.pdf]
